# In silico prediction models for thyroid peroxidase inhibitors and their application to synthetic flavors

**DOI:** 10.1007/s10068-022-01041-y

**Published:** 2022-03-12

**Authors:** Mihyun Seo, Changwon Lim, Hoonjeong Kwon

**Affiliations:** 1 Department of Food and Nutrition, Seoul National University, Seoul, Republic of Korea; 2 Department of Applied Statistics, Chung-Ang University, Seoul, Republic of Korea; 3 Research Institute of Human Ecology, Seoul National University, Seoul, Republic of Korea

**Keywords:** Synthetic flavor, Quantitative structure–activity relationship (QSAR), Thyroid peroxidase inhibitor (TPO), Toxicity prediction, Machine learning

## Abstract

Systematic toxicity tests are often waived for the synthetic flavors as they are added in a very small amount in foods. However, their safety for some endpoints such as endocrine disruption should be concerned as they are likely to be active in low levels. In this case, structure–activity-relationship (SAR) models are good alternatives. In this study, therefore, binary, ternary, and quaternary prediction models were designed using simple or complex machine-learning methods. Overall, hard-voting classifiers outperformed other methods. The test scores for the best binary, ternary, and quaternary models were 0.6635, 0.5083, and 0.5217, respectively. Along with model development, some substructures including primary aromatic amine, (enol)ether, phenol, heterocyclic sulfur, and heterocyclic nitrogen, dominantly occurred in the most highly active compounds. The best predicting models were applied to synthetic flavors, and 22 agents appeared to have a strong inhibitory potential towards TPO activities.

**Supplementary Information:**

The online version contains supplementary material available at 10.1007/s10068-022-01041-y.

## Introduction

Flavors are a type of food additives that are intentionally added to foods in order to enhance or fortify their original flavor. However, since flavors are used only in a small amount and their toxicological data are usually scarce, their safety is often assessed with an initial screening method without properly conducting systematic toxicity tests (MFDS, 2017). Among those methods, threshold of toxicological concern (TTC) method evaluates the safety of chemical compounds and sets an acceptable level of intake based on their structure and exposure levels (World Health Organization et al., 2016). The Joint FAO/WHO Expert Committee on Food Additives (JECFA) categorizes the flavors into three groups in accordance with Cramer class and evaluates their safety based upon TTC method. Cramer class was proposed by Cramer et al*.* in (1976), and it classifies the compounds into three classes based upon their potential of oral toxicity (Cramer et al., 1976). However, concerning that some endpoints such as endocrine disruptors could be active in low doses (Vandenberg et al., 2012; Vandenberg, 2014) and hormonal modulation is becoming a more important health issues nowadays, it is important to deal with those flavors having hormone-modulating activities even if they are used only in small amount. Thyroid hormones, for example, play an important role in many developmental process and regulate metabolic homeostasis (De Coster and van Larebeke, 2012), so the thyroid hormone modulation during developmental stage can lead to serious defect in neurogenesis as well as metabolic disturbance (Gore et al., 2015). Therefore, this study has attempted to conduct a quick screening of potential thyroid peroxidase (TPO)-inhibiting synthetic flavors using in silico prediction models.

Currently, many studies and programs on machine-learning-based quantitative structure–activity relationship (QSAR) and in silico toxicity prediction were conducted (Fan et al., 2018; Idakwo et al., 2018; Jiang et al., 2019; Li et al., 2017; Zhang et al., 2018; Zhang et al., 2020). Results from high-throughput experiments may be employed to build a model. They are available from various databases, such as PubChem or EPA Chemistry Dashboard (Kim et al., 2019; Williams et al., 2017). Tox21, one of these databases, is a collaboration program of several federal agencies in US that aims to improve pre-existing testing strategies and develops many high-throughput screening methods that can be applied to toxicity prediction models (National Toxicology Program, 2020). In many QSAR practices, however, too many features compared to the size of the dataset, and class imbalance have been considered troublesome. Many attempts were made to address these problems, such as dimensionality reduction and under- or over-sampling. A previous study showed that the class-imbalance problem can be solved to some extent by the application of the so-called synthetic minority over-sampling technique (SMOTE) to low-dimensional models (Blagus and Lusa, 2013). In addition, learning methods also play a significant role in model performance. Simple but powerful learning methods, such as support vector machines (SVMs), random forests (RFs), and artificial neural networks (ANNs), demonstrated good performance and were used in many previous QSAR studies (Fan et al., 2018; Fan et al., 2018; Jiang et al., 2019; Li et al., 2014; Li et al., 2017). Recently, many ensemble-learning approaches were also introduced to improve the performance of models (Ai et al., 2019; Sheffield and Judson, 2019; Zhang et al., 2018).

Hence, in this study, prediction models for TPO inhibition were designed using various learning and dimensionality-reduction methods, and SMOTE. In addition, along with the over-sampling technique, multi-categorization of the dataset was attempted to improve the classification performance for minor classes, and their performance was compared with those of binary models. As well as the prediction model development, typical substructures frequently found in highly active compounds were analyzed using substructure frequency analysis (Jensen et al., 2007) and food-related compounds such as food additives or contact materials that feature active substructures were identified. The best models for each grouping method selected according to their test scores were applied to synthetic flavors currently used in South Korea. The flavors predicted by the models in this study to have strong inhibition effects towards TPO were then compared with their Cramer classes.

## Materials and methods

### Data curation and categorization

AC_50_ and IC_50_ values of various TPO inhibitors were collected from The Simmons Lab at the EPA National Center for Computational Toxicology’s Amplex® UltraRed (AUR) assay data, and existing articles (Carvalho et al., 2000; Divi and Doerge, 1996; Habza-Kowalska et al., 2019; Lee, 2015). After removing salts, mixtures, and duplicated ones, 587 data items, including drugs, natural compounds, and environmental chemicals, were used to build machine-learning models. Given that AC_50_ is a relative value that is calculated from each chemical’s maximum inhibition rate, it had to be converted to the absolute IC_50_ value, which is the concentration at which the chemicals inhibit TPO activities to 50% of maximal activity. The conversion is conducted via the following expression (Sebaugh, 2011):

1 $$\log {\text{IC}}_{{50}} = \log {\text{AC}}_{{50}} \times \left( {\frac{{a - 50\%\, {\text{response}}}}{{50\% \,{\text{response}} - b}}} \right)^{{1/c}}$$

*a*: min response; *b*: max response; *c*: slope factor.

Based on their maximum inhibition and the converted IC_50_ values, the collected and purified data were categorized into two (binary), three (ternary), or four (quaternary) groups. For the binary model, the chemicals that inhibited TPO activities more than or equal to 20% at the highest concentration were defined as group ‘A’ (active) as maintained by EPA (Friedman et al., 2016), and the remaining chemicals whose maximum inhibition rate was less than 20% were labeled as group ‘C’ (inactive). For the ternary model, the chemicals with maximum inhibition rate higher than 50%—in which their IC_50_ could be calculated, between 20 and 50%, and less than 20% were labeled as group ‘A’, ‘B’, and ‘C’, respectively. The chemicals in group ‘A’ in the ternary model were subdivided into group ‘A1’ and ‘A2’ in the quaternary model based on their IC_50_ values; the chemicals whose IC_50_ values were lower than or equal to 10 μM were defined as group ‘A1’, and those with IC_50_ higher than 10 μM as group ‘A2’, which was based upon previous studies on enzyme inhibition (Auld et al., 2008; MFDS, 2013; Lindström et al., 2019).

### Feature generation and structure

Molecular descriptors and fingerprints (FPs) were used as features in the machine-learning models. In this study, a ‘topology-substructure concatenated FP’ was employed to consider various features and prevent underfitting. It consists of a concatenation of each topological FP with a substructure key-based FP to consider the atomic connectivity, substructures, and their interactions within a molecule. RDKit, Morgan, and Atom Pair Count (APC) FPs were selected for topological FP, and the substructure count and ToxPrint FP were selected for the substructure key-based FP. RDKit and Morgan FPs were calculated using the RDKit library (Landrum, 2020), and APC, substructure count FP, and descriptors were calculated with the PaDEL-Descriptor software (Yap, 2011). ToxPrint FP was generated using the ChemoTyper program (Mn-Am, 2020).

### Simple and complex ensemble learning methods

Both simple learning methods (SLMs) and complex ensemble learning methods (ELMs) were used to predict TPO inhibitors. As SLMs, a RF, a SVM, and an ANN were used given that they demonstrated to perform well on QSAR tasks in previous studies. Boosting and voting were utilized for the ELMs; adaptive boosting (AdaB) and extreme gradient boosting (XGB) were employed for boosting; and hard- and soft-voting were used as voting models. In the case of voting classifiers, the four best models among RF, SVM, ANN, AdaB, and XGB were selected for each feature and grouping method. The combinations of the four best models selected for the voting classifiers for each feature and grouping method are listed in Table S1.

### Model selection and evaluation

Through the overall process conducted in this study, the Scikit-Learn library was used for feature processing and machine-learning modeling (Pedregosa et al., 2011). The entire dataset was randomly split into an 80:20 ratio, and the 20% subset was used as a test set subsequently. The ‘stratify’ parameter was applied to retain the compositional consistency of each category in both training and test sets. The number of compounds in each category is presented in Table 1.

**Table 1:** Number of compounds in each subset and their classification criteria

Max inhibition	IC_50_	Binary	Number of compounds		Ternary	Number of compounds		Quaternary	Number of compounds	
Higher than 50%	≤ 10 μM	A	Train	388	A	Train	283	A1	Train	100
									Test	25
	> 10 μM					Test	71	A2	Train	183
			Test	97					Test	46
Between 20 and 50%					B	Train	105	B	Train	105
						Test	26		Test	26
Less than 20%		C	Train	81	C	Train	81	C	Train	81
			Test	21		Test	21		Test	21

A grid search with fivefold cross-validation on the training dataset was conducted for hyperparameter tuning and model validation and to prevent overfitting. The hyperparameter grid of each learning method is given in Table S2. Min–max feature normalization, variance thresholder, feature extraction for dimensionality reduction, SMOTE, and model training steps were implemented for each iteration of the fivefold cross-validation. Dimensionality reduction was conducted to solve the overfitting problem that may occur due to the large number of features. The features whose variance was less than 0.01 were removed. Either principal component analysis (PCA) or linear discriminant analysis (LDA) were applied, and their performance was compared. To address the class-imbalance problem, SMOTE was applied to each model. Through this process, the best combination of hyperparameters for each model was selected, and cross-validation scores (CV scores) were calculated as a F1-score value. F1 score, i.e., the harmonic mean of precision and recall, was employed owing to the class-imbalance problem. The formulae of precision, recall, and F1 score are as follows:

$${\text{Precision = }}\frac{{{\text{TP}}}}{{{\text{TP + FP}}}}$$

$${\text{Recall = }}\frac{{{\text{TP}}}}{{{\text{TP + FN}}}}$$

2 $$F1{\text{ score}} = \frac{{2~ \times ~{\text{Precision}}~ \times ~{\text{Recall}}}}{{{\text{Precision}}~ + ~{\text{Recall}}}}$$

[TP: True positive; FP: False positive; FN: False negative]

The best models for each grouping and FP were selected based on the CV score, and their performance was evaluated by applying the models on the test sets. Then, the best models that produced the highest F1 score on the test set was singled out from each grouping as the final best-performing models. All combinations of models, FPs, and dimensionality-reduction methods (or voting methods) for each grouping method are listed in Table S3. After carrying out model selection and evaluation, the effects of the type of features, feature extraction method, and learning method on model performance were evaluated and compared.

### Analysis of active substructures in TPO inhibitors

The substructures that were frequently seen in the most active groups were analyzed using the substructure frequency analysis method previously introduced by Jensen et al. (2002). The frequency of a substructure in group A1 (the most highly active) and C (inactive) in the quaternary grouping was calculated as $\frac{{\frac{{f_{x} }}{{C_{x} }}}}{{\frac{f}{C}}} = \frac{{f_{x} ~ \times ~C}}{{f~ \times ~C_{x} }}$, where *f*_*x*_ and *C*_*x*_ refer to the number of fragments and compounds in group *x*, respectively, and *f* and *C* refer to the number of fragments and compounds in the whole dataset, respectively. A substructure with a frequency greater than 1.2 in group A1, and simultaneously with a ratio of the frequency of group A1 to that of group C greater than 1.2, was defined as an active substructure.

### Application to flavors

Among 2465 synthetic flavors listed in the Ministry of Food and Drug Safety (MFDS)’s Food Additive Code, 1774 compounds available in the EPA’s CompTox Chemistry Dashboard database were obtained, excluding salts and mixtures. The best classification models selected were applied to these flavoring agents for screening. The Toxtree software (Patlewicz et al., 2008) was employed to classify selected flavor compounds which showed high TPO inhibitory activity, according to Cramer.

## Results and discussion

### Model evaluation and selection

**Fig. 1:**
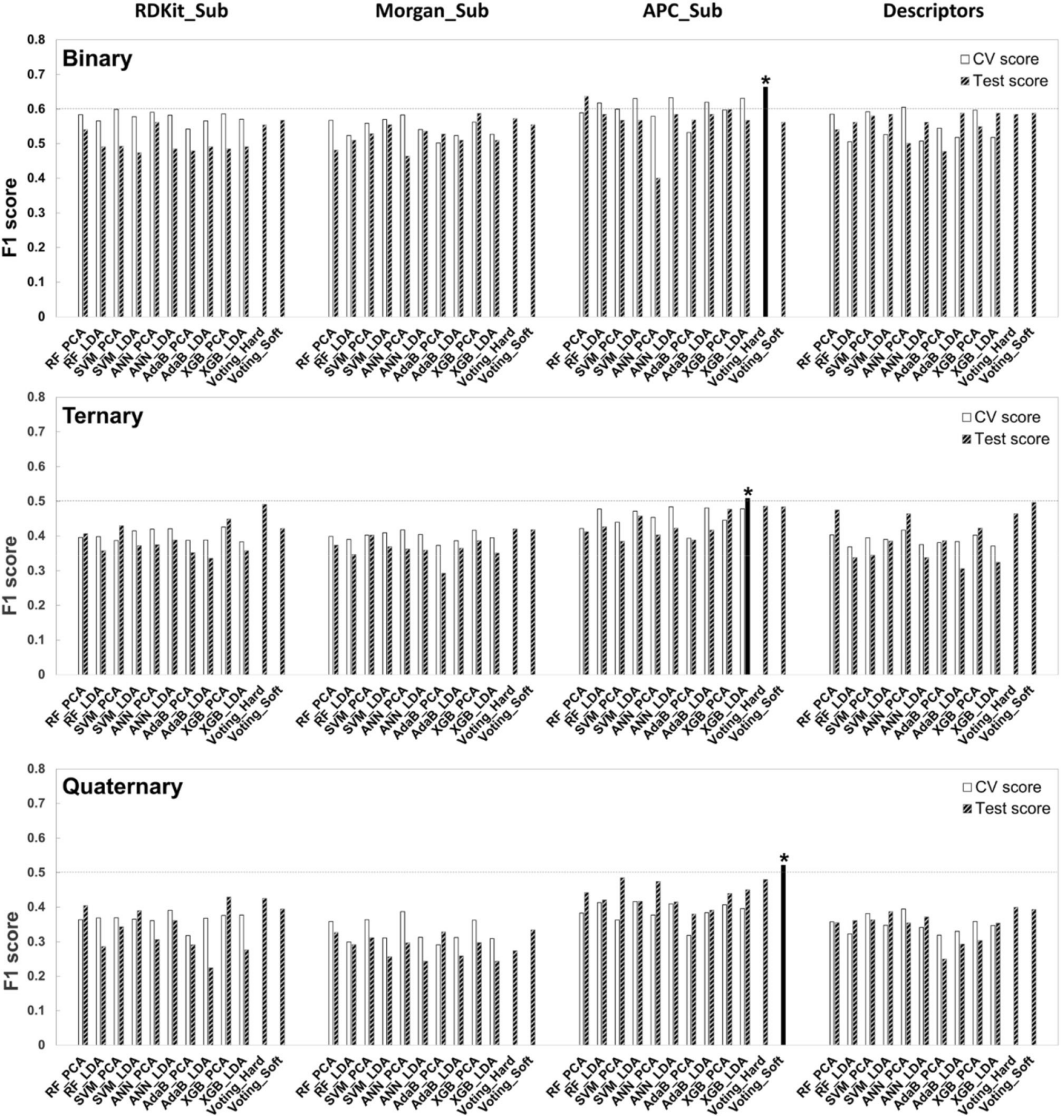
Cross-validation (CV) and test scores for each feature, learning method, and grouping method (binary, ternary, or quaternary). For each grouping method, the black bar marked above with an asterisk indicates the test scores of the best-performing model. Each label is shown in “model name_feature extraction method (or voting methods in voting classifier)” form. [RF: Random forest; SVM: Support vector machine; ANN: Artificial neural network; AdaB: Adaptive boosting; XGB: Extreme gradient boosting; PCA: Principal component analysis; LDA: Linear discriminant analysis]

All the models were evaluated in terms of their test scores. The APC_Sub FP was the best-performing FP, whereas the best-performing models in the binary, ternary, and quaternary models were hard-voting, XGB with LDA, and soft-voting classifier, respectively. The test scores for the aforementioned best models were 0.6635, 0.5083, and 0.5217, respectively (Fig. 1).

In previous studies, Morgan FP is generally known to be one of the best-performing FPs (Idakwo et al., 2018; Riniker and Landrum, 2013). Morgan FP, however, hardly recognizes comprehensive characteristics such as molecular shape or size, and fails to perceive constitutional differences between isomers in large molecules. By contrast, Atom Pair FPs demonstrated to recognize the molecular shape and distinguish the constitutional differences better (Capecchi et al., 2020). Therefore, in this study, APC_Sub FP performed well for molecular shape, substructures and their interactions were better recognized in APC_Sub FP than in Morgan_Sub FP.

To compare the performance of the models by feature extraction (PCA or LDA) or learning methods (RF, SVM, ANN, AdaB, XGB, or Voting), the CV and test scores of each feature extraction or learning methods were pooled and averaged to evaluate each method. For the feature extraction methods, there was no remarkable difference in the performance between the models using PCA and LDA, which is contrary to the expectation that LDA would outperform PCA when it comes to the classification model (Figure S1). Although LDA is generally considered to be a better feature extraction method for classification tasks, PCA may outperform LDA when the dataset is small (Martinez and Kak, 2001). Additionally, it can be inferred that LDA did not perform much better than PCA, given that the categorization of toxicity in this study was done from continuous maximum inhibition (%) and IC_50_ values. Therefore, it seems that no significant difference in performance between LDA and PCA was observed because of the small size of the dataset and the linear characteristic of toxicity class data.

Meanwhile, learning methods were shown to have a relatively greater impact on model performance than feature extraction methods (Figure S2).

For all groupings, hard-voting classifiers remarkably improved the model performance, especially in the ternary and quaternary models. In the case of the ternary model, the hard-voting classifier showed approximately 16, 15, and 14% enhancements in average model performance compared to SLMs–RF, SVM, and ANN–respectively. For quaternary models, the hard-voting classifiers showed 15, 13, and 14% improvement with respect to the aforementioned SLMs–RF, SVM, and ANN–respectively. Among all the models, the worst-performing learning method was the AdaB classifier. For the binary, ternary, and quaternary models, the model performance of the hard-voting classifier was 13, 23, and 31% greater than that of AdaB, respectively. Compared to a previous study that has developed binary models for TPO inhibitors (Rosenberg et al., 2017), the result shows that the binary models in this study have similar levels of F1 scores to those of previous study by employing hard voting classifiers, in spite of smaller dataset.

**Fig. 2:**
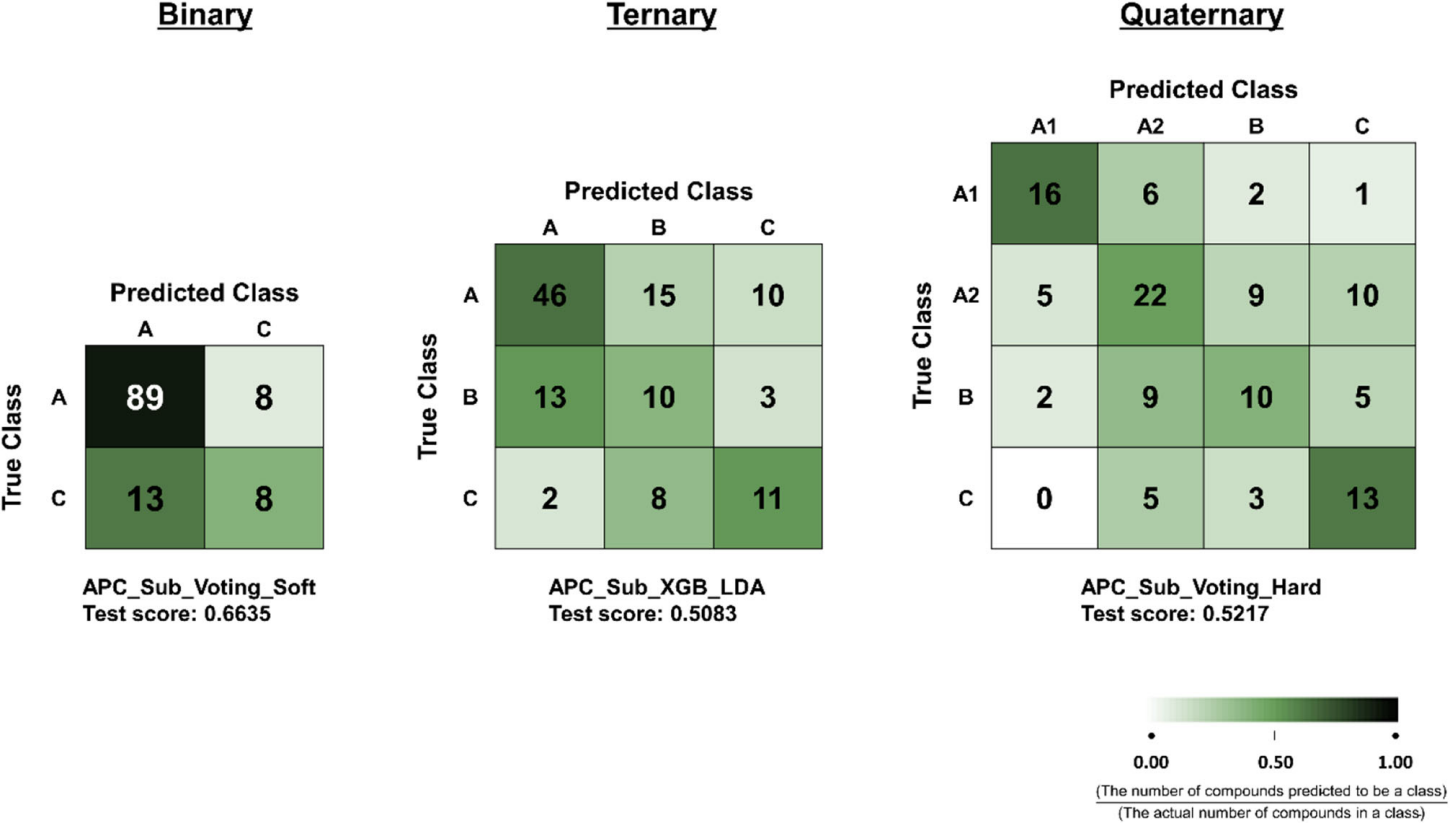
Confusion matrices for the best-performing models of each grouping. The color intensity of each cell represents the proportion of the number of compounds predicted to be a class with respect to the actual number of compounds in a class

Along with F1 scores, confusion matrices for the best-performing models indicated in Fig. 1 were generated to evaluate the classification performance in detail (Fig. 2). Based on confusion matrices, negative data were better classified in the ternary and quaternary models, even though the test score was the highest in the binary model. The specificities of the best binary, ternary, and quaternary models were 0.3810, 0.5238, and 0.6190, respectively. Furthermore, continuous toxicity data such as IC_50_ would allow more precise prediction of toxicity strength of substances, but they are more difficult to be obtained in large numbers. Therefore, this work is significant in that multi-classification models were developed with small numbers of IC_50_ data by adopting ensemble learning methods.

### Active substructure analysis

An active substructure analysis was conducted using the substructure frequency analysis method. The active substructures included amines (especially primary aromatic amines and heterocyclic nitrogen), sulfur-containing compounds (carbothioic S-ester, thioenolether, thiocyanate, sulfenic derivatives, carbodithioic ester, heterocyclic sulfur, etc.), phenols, ethers (enolether and thioenolether), and vinylogous compounds. The most frequent and dominant substructure was primary aromatic amine with a frequency ratio of 35.90, meaning that it appears 35.90 times more frequently in group A1 than in group C. The inhibition mechanism of aromatic amines towards TPO was previously demonstrated by Doerge et al*.* (1994) The aromatic amines inhibit TPO activity by interacting with the compound I (an oxyferryl cation radical of iron-containing heme cofactor) instead of iodine, i.e., an endogenous substrate of TPO (Doerge and Decker, 1994).

Other frequent substructures were enol (enolether), sulfenic derivatives, and phenols, for which the frequency ratios were 9.79 (8.16), 8.16, and 4.45, respectively. Some examples of active compounds in group A1 and their corresponding active substructures are listed in Table 2. Among these highly potential compounds, food-related chemicals can be pointed out, including natural food substances, food additives or contact materials, and pesticides. L-ascorbic acid, L-tryptophan, and polyphenols such as quercetin and genistein are typical examples of natural food substances that inhibit TPO activities. Food additives or contact materials such as indole (synthetic flavor) or 4,4’-methylenedianiline (food contact material) were also found to be highly active. In addition, some sulfur-containing or organophosphorus pesticides were included in the A1 group. While the frequency of phosphate substructures in group A1 was less than 1.2, their frequency in group A2 was 1.34. Furthermore, the frequency ratios of groups A1 and A2 to group C were 2.72 and 3.14, respectively. Hence, it can be stated that phosphate substructures in organophosphorus pesticides could also be considered as active substructures.

**Table 2:** Examples of compounds in the highest activity group (group A1) shown with active substructures studied by means of substructure frequency analysis (depicted in red)

Name of compounds	Structures of compounds and corresponding active substructure	Purpose of use or distribution in nature	Ref
4′4′-methylenedianiline		Food contact material	(U.S. Food and Drug Administration, 2020a)
Quercetin		A flavonoid (flavonol) in edible plants (e.g., onion)	(Chandra, 2010)
Genistein		A flavonoid (isoflavone) in edible plants (e.g., soybean)	(Chandra, 2010)
L-ascorbic acid		Natural food substance; food additive	(U.S. Food and Drug Administration, 2020b)
L-Tryptophan		Natural food substance (amino acid)	(U.S. Food and Drug Administration, 2020c)
Isoproterenol		Drug; β-adrenergic receptor agonist (analogue of epinephrine)	(U.S. National Library of Medicine, 2020)
Indole		Food additive (flavoring agent)	(Food and Agriculture Organization of the United States (FAO), 2020)
2-Mercaptobenzothioazole		Pesticide	(United States Environmental Protection Agency (EPA), 2020)
Phenmedipham		Pesticide	(European Commission, 2020)
Azinphos-methyl		Pesticide	(European Commission, 2020)
Dimethoate		Pesticide	(European Commission, 2020)
Malathion		Pesticide	(European Commission, 2020)

### Application to flavors

**Fig. 3:**
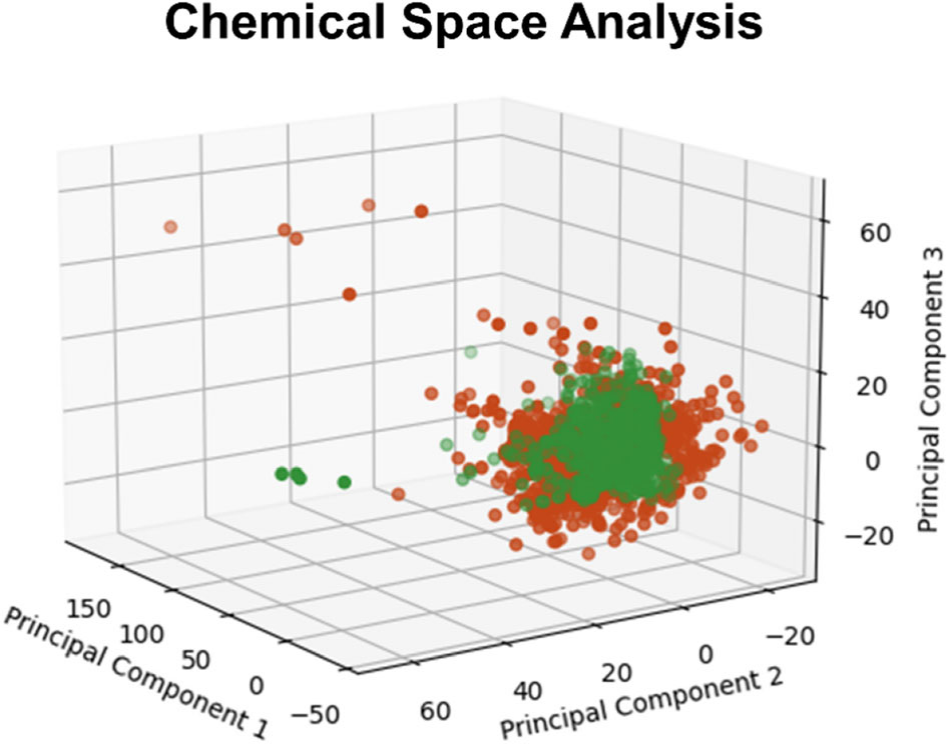
Chemical space analysis between the input data for model development and synthetic flavors (red dots: synthetic flavors; green dots: input data for model development)

In addition to the substructure analysis, 1774 synthetic flavors currently listed in South Korean Food Code were applied to the three best performing models. Before the application, the chemical spaces of these synthetic flavors and of the dataset used for model development were analyzed with three principal components extracted from their molecular descriptors (Fig. 3). Most flavor compounds showed a comparable chemical space with that of the dataset employed in this study.

As a result, 22 out of 1774 were found to be in the most active group both in the ternary (group A) and quaternary (group A1) models and active in the binary model (group A). The molecular structures of these 22 compounds include primary aromatic amines, vinylogous carbonyls, and sulfur-containing substances (Table 3). These synthetic flavors, predicted to be highly active by the models, were compared with their Cramer classes predicted by the Toxtree software.

**Table 3:** Synthetic flavors found to be active in the predictive models and their corresponding Cramer classes predicted by the Toxtree software

Name of compound	Structure	Cramer class^a^		
		Class I	Class II	Class III
Indole				✓
Pyrrole				✓
Butyl anthranilate		✓		
Linalyl anthranilate		✓		
(+)-Cedrol				✓
Geranyl tiglate		✓		
Allyl Ionone		✓		
Bis(2-methyl-3-furyl)disulfide				✓
(3aR)-(+)-Sclareolide				✓
Maltol isobutyrate				✓
Cedr-8(15)-en-4-ol		✓		
2’-Aminoacetophenone		✓		
Patchouli alcohol				✓
Elemol				✓
Dibenzyl disulfide				✓
Difurfuryl disulfide				✓
2-Furfurylthio-3-methylpyrazine				✓
Furfuryl-2-methyl-3-furyl disulfide				✓
8,8-diethoxy-2,6-dimethyl-2-Octanol				✓
Viridiflorol				✓
Caryolan-1-ol				✓
L-Methionylglycine				✓

^a^Cramer classes are defined as follows:

Class I: Chemical structures with small potential of oral toxicity (low class)

Class II: Chemical structures that are more potentially toxic than Class I substances, but without typical structures suggestive of toxicity (intermediate class)

Class III: Chemical structures that have no solid evidence of safety or may even have functional groups that suggest strong toxicity or reactivity (high class)

As synthetic flavors are added to foods only in a small amount, they are often regulated with threshold of toxicological concern (TTC) method, which is based on the chemical structures of substances. In JECFA, synthetic flavors are categorized into one of the structural classes proposed by Cramer et al. (1976) and their structures, metabolic fate and intake levels are assessed (Joint FAO/WHO Expert Committee on Food Additives, 2002). Cramer decision tree classifies compounds into three groups–Class I (low concern of oral toxicity), Class II (moderate concern of oral toxicity), and Class III (high concern of oral toxicity), according to the predicted intensity of their oral toxicity (Cramer et al., 1976; Roberts et al., 2015). When the Cramer class of these 22 active flavor compounds was determined using the Toxtree software, 16 of them were classified into Class III, while 6 were classified into Class I. These six substances were butyl anthranilate, linalyl anthranilate, geranly tiglate, allyl ionone, cedr-8(15)-en-4-ol, and 2’-aminoacetophenone. Aniline substructures stand out among the compounds that show high TPO inhibitory potential but low overall oral toxicity potential. The TPO-inhibiting effect and mechanism of action of substances with aromatic amines are clearly reported (Doerge et al., 1994). Moreover, as it is known that endogenous hormones work at very low concentrations in the body, even a slight modulation in the endocrine system in their low-dose range might significantly change the hormonal effect (Vandenberg et al., 2012).

Although the prediction models in this study are based on in vitro experimental data, it may be necessary to ameliorate Cramer’s decision tree with recent toxicity result in light of the current situation that hormonal modulation is becoming a more important health issue. Further, in order to support the findings of this study, a follow-up study is necessary that determines whether the 22 flavors actually have TPO-inhibition activity through a laboratory experiment.

Summing up, the present study raises the possibility that some synthetic flavors may have a health effect on TPO activities through in silico toxicity prediction models. Binary, ternary, and quaternary prediction models were designed using various machine-learning methods. The best models for the binary, ternary, and quaternary models were ‘hard-voting classifier with APC_Sub FP’, ‘XGB with APC_Sub FP and LDA’, and ‘soft-voting classifier with APC_Sub FP’, respectively, and the F1 scores on the test set for each best model were 0.6635, 0.5083, and 0.5217, respectively. Although the test score was the highest in the binary model, the minor class (group C) was better predicted in the ternary and quaternary models. However, it should be concerned that these prediction models are based on in vitro experimental data, which hardly considers toxicokinetics of chemicals in the body.

The most frequent and dominant substructures within the highly active compounds (group A1) were primary aromatic amines, sulfur-containing, phenols, vinylogous compounds, and phosphates. When the best models selected from each grouping method were applied to 1774 synthetic flavors listed in South Korea, 22 out of 1774 agents were predicted to show inhibitory activity toward TPO. Sixteen out of 22 substances belonged to Cramer Class III, while six were Class I. Among these 6 compounds, which were predicted to have high TPO inhibitory activity but classified as low oral toxicity in Cramer class, three compounds had aniline substructures, butyl anthranilate, linalyl anthranilate, and 2’-aminoacetophenone, suggesting a revision of Cramer class to encompass hormonal modulation.

To suggest, follow-up study on the prediction model that involves the toxicokinetics of the TPO-inhibiting substances and on the determination of the 22 active flavors through actual laboratory experiments will support the major findings in this study.

## Supplementary Information

Below is the link to the electronic supplementary material.

Supplementary file1 (CSV 6752 kb)

Supplementary file2 (CSV 1291 kb)

Supplementary file3 (CSV 977 kb)

Supplementary file4 (CSV 2750 kb)

Supplementary file5 (XLSX 7123 kb)

Supplementary file6 (IPYNB 111 kb)

Supplementary file7 (IPYNB 132 kb)

Supplementary file8 (PKL 1499 kb)

Supplementary file9 (PKL 685 kb)

Supplementary file10 (IPYNB 123 kb)

Supplementary file11 (IPYNB 128 kb)

Supplementary file12 (IPYNB 102 kb)

Supplementary file13 (IPYNB 108 kb)

Supplementary file14 (PKL 68213 kb)

Supplementary file15 (PKL 290 kb)

Supplementary file16 (PKL 1402 kb)

Supplementary file17 (PKL 37412 kb)

Supplementary file18 (PKL 155 kb)

Supplementary file19 (PKL 81148 kb)

Supplementary file20 (XLSX 1856 kb)

Supplementary file21 (XLSX 1388 kb)

Supplementary file22 (XLSX 4352 kb)

Supplementary file23 (PKL 77 kb)

Supplementary file24 (PKL 1381 kb)

Supplementary file25 (PKL 32339 kb)

Supplementary file26 (TXT 23 kb)

Supplementary file27 (DOCX 552 kb)

Supplementary file28 (DOCX 36 kb)
