# Supplementary material for: In silico prediction models for thyroid peroxidase inhibitors and their application to synthetic flavors
Source: Food Sci Biotechnol. 2022 Mar 12;31(4):483–95. doi: 10.1007/s10068-022-01041-y (PMC8994803; doi:10.1007/s10068-022-01041-y)
Supplement: Supplementary file 27 — Supplementary file27 (DOCX 552 kb) [file 10068_2022_1041_MOESM27_ESM.docx]

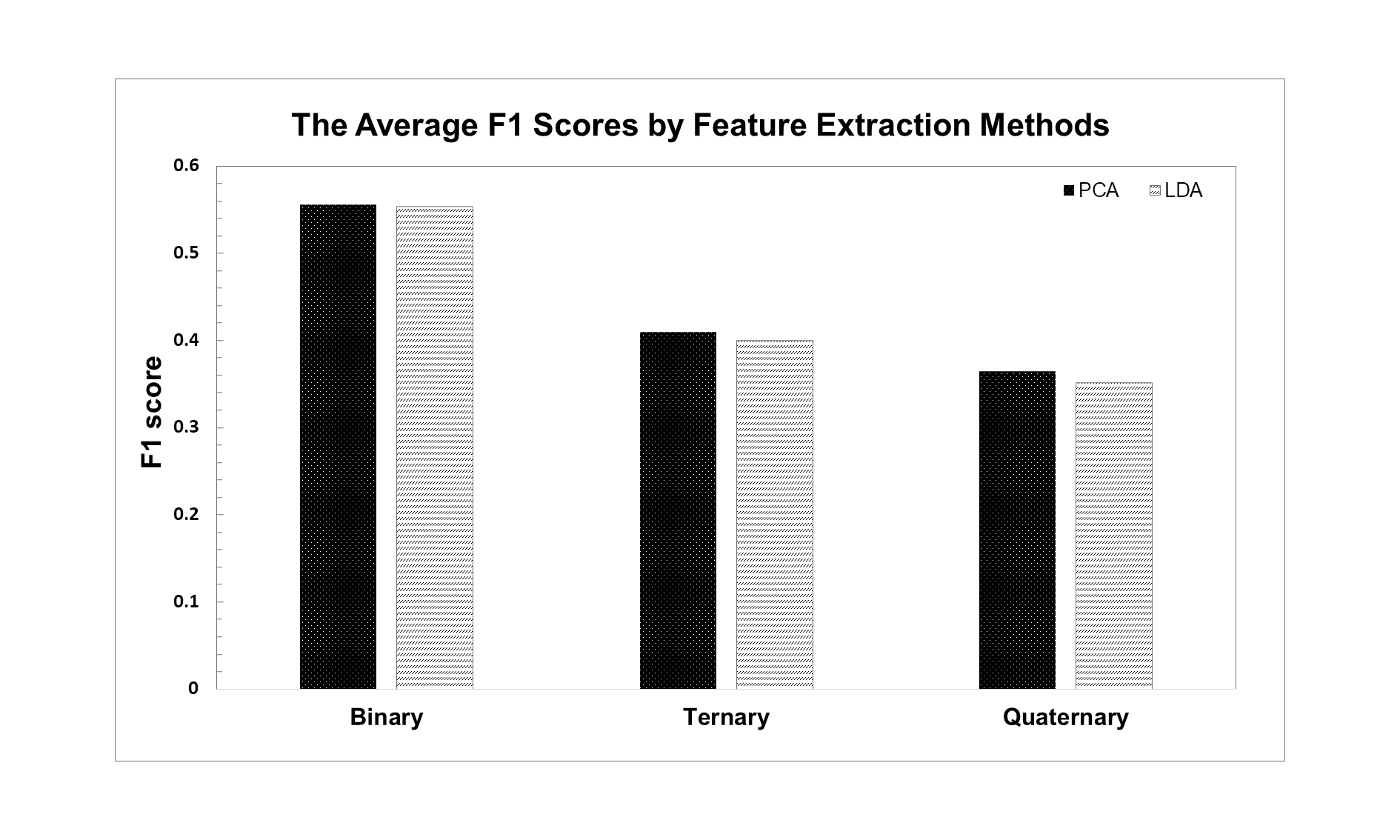


**Figure S1.** F1 scores of each grouping by feature extraction method. No remarkable difference was observed in the performance between the models using PCA and LDA, which does not coincide with the general knowledge that LDA is the better feature extraction method in classification tasks.


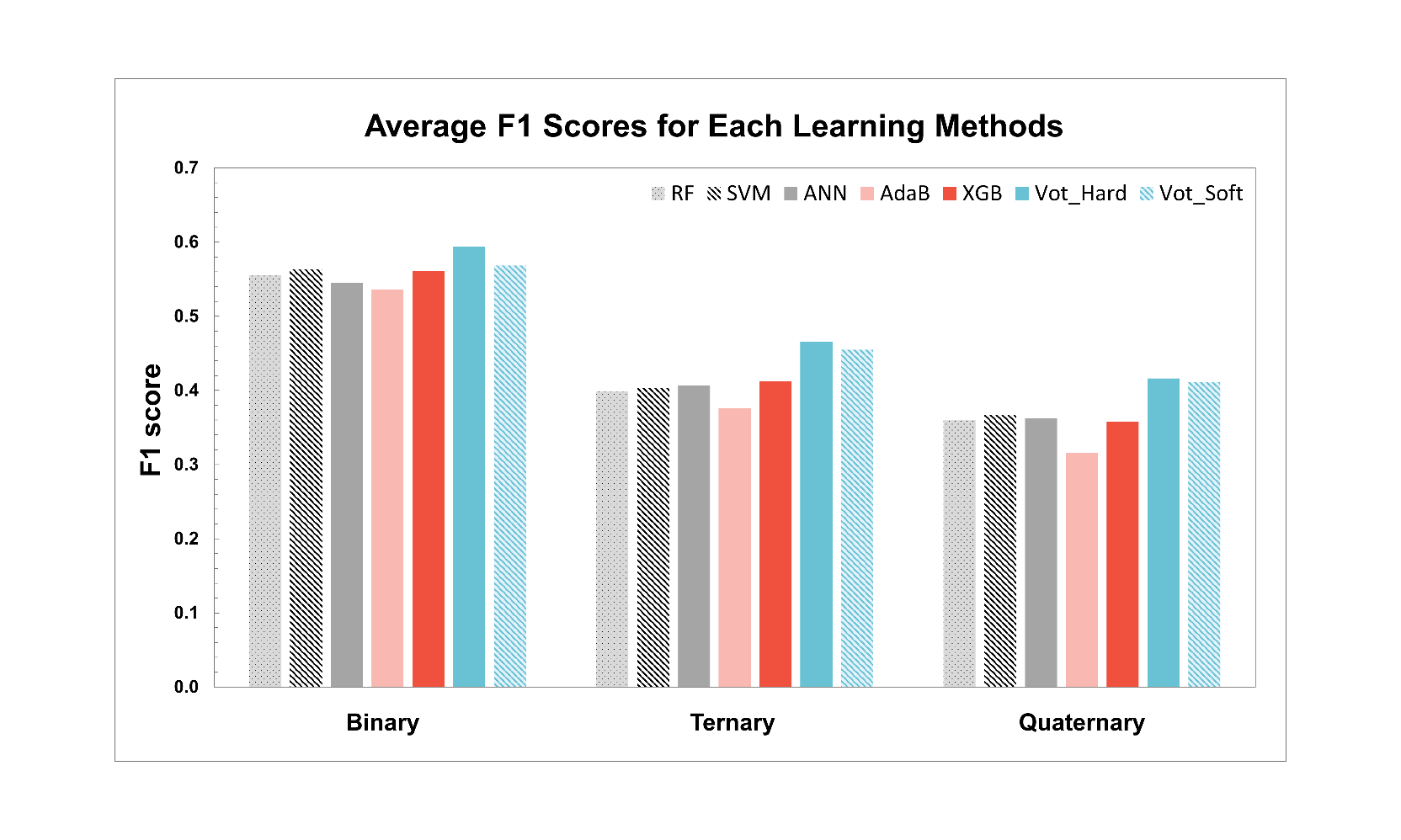


**Figure S2**. F1 scores for each grouping by learning methods. Hard-voting classifiers improved the model performance in every grouping, especially in the ternary and quaternary models. [RF: Random forest; SVM: Support vector machine; ANN: Artificial neural networks; AdaB: Adaptive boosting; XGB: Extreme gradient boosting; Vot_Hard: Hard-voting; Vot_Soft: Soft-voting]
