# Supplementary material for: In silico prediction models for thyroid peroxidase inhibitors and their application to synthetic flavors
Source: Food Sci Biotechnol. 2022 Mar 12;31(4):483–95. doi: 10.1007/s10068-022-01041-y (PMC8994803; doi:10.1007/s10068-022-01041-y)
Supplement: Supplementary file 28 — Supplementary file28 (DOCX 36 kb) [file 10068_2022_1041_MOESM28_ESM.docx]

**Table S1.** The combinations of four machine learning models used in voting classifiers for each grouping method.

| **Binary** | | | | |
| --- | --- | --- | --- | --- |
|  | **Model 1** | **Model 2** | **Model 3** | **Model 4** |
| **RDKit_Sub FP** | ANN_PCA | RF_PCA | SVM_PCA | XGB_LDA |
| **Morgan_Sub FP** | XGB_PCA | SVM_LDA | ANN_LDA | SVM_PCA |
| **APC_Sub FP** | RF_PCA | XGB_PCA | ANN_LDA | RF_LDA |
| **Descriptors** | AdaB_LDA | XGB_LDA | SVM_LDA | SVM_PCA |
|  | | | | |
| **Ternary** | | | | |
|  | **Model 1** | **Model 2** | **Model 3** | **Model 4** |
| **RDKit_Sub FP** | XGB_PCA | SVM_PCA | RF_PCA | SVM_LDA |
| **Morgan_Sub FP** | SVM_PCA | XGB_PCA | RF_PCA | SVM_LDA |
| **APC_Sub FP** | XGB_LDA | SVM_LDA | XGB_PCA | RF_LDA |
| **Descriptors** | RF_PCA | ANN_PCA | XGB_PCA | SVM_LDA |
|  | | | | |
| **Quaternary** | | | | |
|  | **Model 1** | **Model 2** | **Model 3** | **Model 4** |
| **RDKit_Sub FP** | XGB_PCA | RF_PCA | SVM_LDA | ANN_LDA |
| **Morgan_Sub FP** | AdaB_PCA | RF_PCA | SVM_PCA | RF_LDA |
| **APC_Sub FP** | SVM_PCA | ANN_PCA | XGB_LDA | RF_PCA |
| **Descriptors** | SVM_LDA | ANN_LDA | SVM_PCA | RF_LDA |

All the model names are presented in the form of ‘learning method_feature extraction method’.

RF: random forest; SVM: support vector machine; ANN: artificial neural network; AdaB: adaptive boosting; XGB: extreme gradient boosting;

**Table S2.** The hyperparameter grid used in the 5-fold cross-validated grid search for each model.

|  | **Hyperparameter grid** | | | | |
| --- | --- | --- | --- | --- | --- |
| **PCA** | | | | | |
| *n_components* | 1 | 2 | 3 |  |  |
| **LDA** | | | | | |
| *n_components*^a^ | 1 | 2 | 3 |  |  |
| **Random forest** | | | | | |
| *n_estimators* | 200 | 400 | 800 | 1600 | 3200 |
| *max_depth* | 5 | 10 | 20 | 40 | 80 |
| **Support vector machine** | | | | | |
| *C* | 0.01 | 0.1 | 1 | 10 |  |
| *gamma* | 0.01 | 0.1 | 1 | 10 |  |
| *kernel* | ‘rbf' | ‘poly’ |  |  |  |
| *degree*  *(when kernel = ‘poly’)* | 1 | 2 | 3 |  |  |
| **Artificial neural network** | | | | | |
| *hidden_layer_sizes* |  |  |  |  |  |
| the number of nodes^b^ | 5 | 10 | 50 | 100 | 200 |
| *activation* | ‘relu’ | ‘logistic’ | ‘tanh’ | ‘identity’ |  |
| *solver* | ‘lbfgs’ | ‘adam’ | ‘sgd’ |  |  |
| *alpha^c^* | 0.00001 | 0.001 | 0.1 | 10 |  |
| **Adaptive boosting** | | | | | |
| *base_estimator* | Decision tree (DT) | | | | |
| *n_estimators*^c^ | 200 | 400 | 800 | 1600 | 3200 |
| *learning_rate*^c^ | 0.2 | 0.4 | 0.6 | 0.8 | 1 |
| **Extreme gradient boosting** | | | | | |
| *n_estimators*^c^ | 200 | 400 | 800 | 1600 | 3200 |
| *max_depth*^c^ | 5 | 10 | 20 | 40 | 80 |
| *learning_rate*^c^ | 0.2 | 0.4 | 0.6 | 0.8 | 1 |
| *colsample_bytree*^c^ | 0.1 | 0.2 | 0.4 | 0.6 | 0.8 |
| *reg_alpha*^c^ | 0 | 0.01 | 0.1 | 1 | 10 |
| *reg_lambda*^c^ | 0 | 0.01 | 0.1 | 1 | 10 |

^a^ The maximum number of components in LDA is min[(the number of classes) - 1, (the number of features)].

^b^ The optimal number of nodes was given by grid search and then the number of layers was adjusted.

^c^ After obtaining the optimal hyperparameters using random search, fine-tuning was conducted via grid search.

**Table S3.** 144 combinations of fingerprints (FPs), learning methods and dimensionality reduction or voting method for each grouping.

|  | | | **RDKit_Sub FP** | **Morgan_Sub FP** | **APC_Sub FP** | **Descriptor** |  | | **RDKit_Sub FP** | **Morgan_Sub FP** | **APC_Sub FP** | **Descriptor** |
| --- | --- | --- | --- | --- | --- | --- | --- | --- | --- | --- | --- | --- |
| **Binary** | **RF** | **PCA** | RDK_Sub_ RF_PCA | Morg_Sub_ RF_PCA | APC_Sub_ RF_PCA | Descriptor_ RF_PCA | **AdaB** | **PCA** | RDK_Sub_ AdaB_PCA | Morg_Sub_ AdaB_PCA | APC_Sub_ AdaB_PCA | Descriptor_ AdaB_PCA |
|  |  | **LDA** | RDK_Sub_ RF_LDA | Morg_Sub_ RF_LDA | APC_Sub_ RF_LDA | Descriptor_ RF_LDA |  | **LDA** | RDK_Sub_ AdaB_LDA | Morg_Sub_ AdaB_LDA | APC_Sub_  AdaB_LDA | Descriptor_ AdaB_LDA |
|  | **SVM** | **PCA** | RDK_Sub_ SVM_PCA | Morg_Sub_ SVM_PCA | APC_Sub_ SVM_PCA | Descriptor_ SVM_PCA | **XGB** | **PCA** | RDK_Sub_ XGB_PCA | Morg_Sub_  XGB_PCA | APC_Sub_  XGB_PCA | Descriptor_ XGB_PCA |
|  |  | **LDA** | RDK_Sub_ SVM_LDA | Morg_Sub_ SVM_LDA | APC_Sub_ SVM_LDA | Descriptor_ SVM_LDA |  | **LDA** | RDK_Sub_ XGB_LDA | Morg_Sub_ XGB_LDA | APC_Sub_  XGB_LDA | Descriptor_  XGB_LDA |
|  | **ANN** | **PCA** | RDK_Sub_ ANN_PCA | Morg_Sub_ ANN_PCA | APC_Sub_ ANN_PCA | Descriptor_ ANN_PCA | **Vot** | **Hard** | RDK_Sub_  Voting_Hard | Morg_Sub_  Voting_Hard | APC_Sub_  Voting_Hard | Descriptor_  Voingt_Hard |
|  |  | **LDA** | RDK_Sub_ ANN_LDA | Morg_Sub_ ANN_LDA | APC_Sub_ ANN_LDA | Descriptor_ ANN_LDA |  | **Soft** | RDK_Sub_ Voting_Soft | Morg_Sub_  Voting_Soft | APC_Sub_  Voting_Soft | Descriptor  _Voting_Soft |
|  |  | |  |  |  |  |  | |  |  |  |  |
|  | | | **RDKit_Sub FP** | **Morgan_Sub FP** | **APC_Sub FP** | **Descriptor** |  | | **RDKit_Sub FP** | **Morgan_Sub FP** | **APC_Sub FP** | **Descriptor** |
| **Ternary** | **RF** | **PCA** | RDK_Sub_ RF_PCA | Morg_Sub_ RF_PCA | APC_Sub_ RF_PCA | Descriptor_ RF_PCA | **AdaB** | **PCA** | RDK_Sub_ AdaB_PCA | Morg_Sub_ AdaB_PCA | APC_Sub_ AdaB_PCA | Descriptor_ AdaB_PCA |
|  |  | **LDA** | RDK_Sub_ RF_LDA | Morg_Sub_ RF_LDA | APC_Sub_ RF_LDA | Descriptor_ RF_LDA |  | **LDA** | RDK_Sub_ AdaB_LDA | Morg_Sub_ AdaB_LDA | APC_Sub_  AdaB_LDA | Descriptor_ AdaB_LDA |
|  | **SVM** | **PCA** | RDK_Sub_ SVM_PCA | Morg_Sub_ SVM_PCA | APC_Sub_ SVM_PCA | Descriptor_ SVM_PCA | **XGB** | **PCA** | RDK_Sub_ XGB_PCA | Morg_Sub_  XGB_PCA | APC_Sub_  XGB_PCA | Descriptor_ XGB_PCA |
|  |  | **LDA** | RDK_Sub_ SVM_LDA | Morg_Sub_ SVM_LDA | APC_Sub_ SVM_LDA | Descriptor_ SVM_LDA |  | **LDA** | RDK_Sub_ XGB_LDA | Morg_Sub_ XGB_LDA | APC_Sub_  XGB_LDA | Descriptor_  XGB_LDA |
|  | **ANN** | **PCA** | RDK_Sub_ ANN_PCA | Morg_Sub_ ANN_PCA | APC_Sub_ ANN_PCA | Descriptor_ ANN_PCA | **Vot** | **Hard** | RDK_Sub_  Voting_Hard | Morg_Sub_  Voting_Hard | APC_Sub_  Voting_Hard | Descriptor_  Voingt_Hard |
|  |  | **LDA** | RDK_Sub_ ANN_LDA | Morg_Sub_ ANN_LDA | APC_Sub_ ANN_LDA | Descriptor_ ANN_LDA |  | **Soft** | RDK_Sub_ Voting_Soft | Morg_Sub_  Voting_Soft | APC_Sub_  Voting_Soft | Descriptor  _Voting_Soft |
|  |  | |  |  |  |  |  | |  |  |  |  |
|  | | | **RDKit_Sub FP** | **Morgan_Sub FP** | **APC_Sub FP** | **Descriptor** |  | | **RDKit_Sub FP** | **Morgan_Sub FP** | **APC_Sub FP** | **Descriptor** |
| **Quaternary** | **RF** | **PCA** | RDK_Sub_ RF_PCA | Morg_Sub_ RF_PCA | APC_Sub_ RF_PCA | Descriptor_ RF_PCA | **AdaB** | **PCA** | RDK_Sub_ AdaB_PCA | Morg_Sub_ AdaB_PCA | APC_Sub_ AdaB_PCA | Descriptor_ AdaB_PCA |
|  |  | **LDA** | RDK_Sub_ RF_LDA | Morg_Sub_ RF_LDA | APC_Sub_ RF_LDA | Descriptor_ RF_LDA |  | **LDA** | RDK_Sub_ AdaB_LDA | Morg_Sub_ AdaB_LDA | APC_Sub_  AdaB_LDA | Descriptor_ AdaB_LDA |
|  | **SVM** | **PCA** | RDK_Sub_ SVM_PCA | Morg_Sub_ SVM_PCA | APC_Sub_ SVM_PCA | Descriptor_ SVM_PCA | **XGB** | **PCA** | RDK_Sub_ XGB_PCA | Morg_Sub_  XGB_PCA | APC_Sub_  XGB_PCA | Descriptor_ XGB_PCA |
|  |  | **LDA** | RDK_Sub_ SVM_LDA | Morg_Sub_ SVM_LDA | APC_Sub_ SVM_LDA | Descriptor_ SVM_LDA |  | **LDA** | RDK_Sub_ XGB_LDA | Morg_Sub_ XGB_LDA | APC_Sub_  XGB_LDA | Descriptor_  XGB_LDA |
|  | **ANN** | **PCA** | RDK_Sub_ ANN_PCA | Morg_Sub_ ANN_PCA | APC_Sub_ ANN_PCA | Descriptor_ ANN_PCA | **Vot** | **Hard** | RDK_Sub_  Voting_Hard | Morg_Sub_  Voting_Hard | APC_Sub_  Voting_Hard | Descriptor_  Voingt_Hard |
|  |  | **LDA** | RDK_Sub_ ANN_LDA | Morg_Sub_ ANN_PCA | APC_Sub_ ANN_LDA | Descriptor_ ANN_LDA |  | **Soft** | RDK_Sub_ Voting_Soft | Morg_Sub_  Voting_Soft | APC_Sub_  Voting_Soft | Descriptor  _Voting_Soft |

RF: Random forest; SVM: Support vector machine; ANN: Artificial neural network; AdaB: Adaptive Boosting; XGB: Extreme Gradient Boosting; Vot: Voting
